# Usability Testing of a Hair Apposition Device for Scalp Laceration: A Manikin Simulation Study

**DOI:** 10.1155/emmi/5559198

**Published:** 2026-03-17

**Authors:** Hyun Soo Kim, Dongbum Suh, Dae Kon Kim, Jin Hee Lee, Hyuksool Kwon, Chulmin Ha, Hyoung Ju Lee

**Affiliations:** ^1^ Department of Emergency Medicine, Hanil General Hospital, Seoul, 01450, Republic of Korea; ^2^ Department of Emergency Medicine, Seoul National University Bundang Hospital, Seongnam, 13620, Republic of Korea, snubh.org; ^3^ Department of Emergency Medicine, College of Medicine, Seoul National University, Seoul, 03080, Republic of Korea, snu.ac.kr; ^4^ Disaster Medicine Research Center, Seoul National University Medical Research Center, Seoul, 03080, Republic of Korea, snu.ac.kr

**Keywords:** hair apposition technique, scalp laceration, simulation study, usability testing

## Abstract

**Objectives:**

The hair apposition technique (HAT) is a method for closing scalp lacerations by twisting adjacent hair strands, which may require more than one operator. The hair apposition device (HADev), a prototype, was developed to enable single‐operator use by integrating a comb, hair clips, and an anchoring system. Accordingly, the objective of this study was to evaluate the usability of HADev.

**Methods:**

Study participants were medical professionals with prior experience in HAT. Following a 30‐min lecture and practice session, participants performed HAT using HADev on manikins with short (8 cm) and long (20 cm) hair. Each participant completed two procedures for each hair type. Participants were randomized according to the sequence of hair length. The success rate and procedure time were recorded. Questionnaires were administered to assess the perceived difficulty and satisfaction.

**Results:**

Twenty participants (13 physicians and 7 residents) completed 40 procedures using HADev, with 20 performed on short‐haired manikins and 20 on long‐haired manikins. For short hair, physicians and residents achieved success rates of 100% and 85.7%, with median procedure times of 55.1 and 52.8 s, respectively. For long hair, physicians and residents achieved success rates of 92.3% and 100%, with median times of 63.0 and 62.1 s, respectively. The median perceived difficulty scores (1 = very easy, 5 = very difficult) were 2 for HAT and 1 for HADev. The median satisfaction scores (1 = excellent, 5 = very poor) were 2 for physicians and 3 for residents with HAT and 1 for both groups with HADev.

**Conclusion:**

HADev demonstrated feasibility for single‐operator use in a simulated environment, with a 95% success rate and procedure time of approximately 1 min for both hair conditions. However, further studies are needed to improve its usability and assess its clinical applicability.

## 1. Introduction

More than 10 million traumatic wounds are treated annually in emergency departments (EDs) across the U.S. [1], with 14% located on the scalp [2]. In 2016, open scalp wounds contributed to approximately 500,000 ED visits nationwide [3]. The anatomical structure of the scalp, characterized by stretched skin tissue overlying the bone, predisposes the scalp to laceration [4].

Standard wound closure methods include sutures, staples, adhesive tape, and tissue adhesives [5]. Staples are suitable wound closure modalities, particularly for managing scalp lacerations [6]. Compared with sutures, staples provide the advantages of faster application and lower associated infection rates [7]. In recent years, tissue adhesives have emerged as promising alternatives to sutures, offering the benefits of simplified application, reduced procedural time, and avoidance of complications associated with traditional surgical sutures [8].

In 2002, Hock et al. first reported the hair apposition technique (HAT) [9], which involves initial wound cleansing using standard procedures, followed by the approximation of 4‒5 hair strands from each wound margin. The selected strands are crossed and twisted once, followed by the application of a single drop of adhesive to secure wound closure. HAT has demonstrated efficacy in terms of complication rates, procedural duration, pain levels, and cosmetic outcomes when compared to standard suturing [10]. From a cost‐effectiveness perspective, HAT demonstrated superior outcomes compared with standard suturing [11].

Histoacryl glue and medical personnel costs constitute the majority of direct expenses associated with HAT, excluding allocated costs. Allocated costs refer to indirect institutional overhead expenses, such as facility maintenance, administrative support, and equipment depreciation, which were excluded from the cost analysis [11]. Although HAT is cost‐saving compared with standard sutures, it may require at least two medical personnel—one to grasp the hair strands and one to apply the glue. This may incur additional human resource costs associated with HAT.

The hair apposition device (HADev), a prototype, was developed to hold hair strands from each side of the wound, enabling a single medical staff member to perform HAT. The objective of this study was to evaluate the usability of HADev in a manikin‐based simulation.

## 2. Methods

### 2.1. Device Design

**FIGURE 1:**
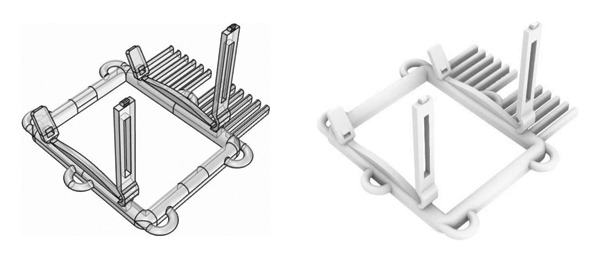
3D design program image of HADev.

**FIGURE 2:**
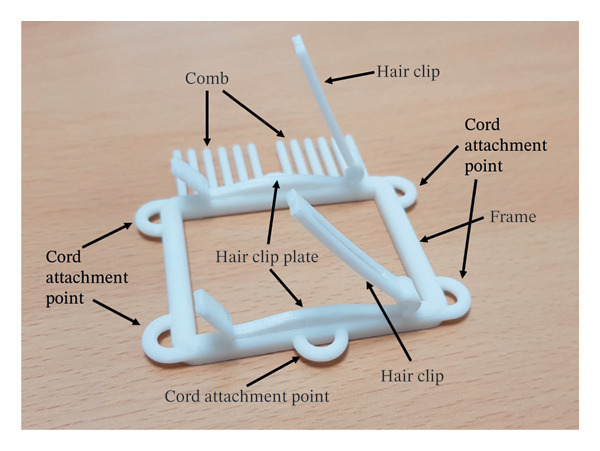
Components of HADev.

HADev was designed using the three‐dimensional (3D) design program Rhinoceros 6 (Rhinoceros 3D, Korea, Figure 1). The main body of HADev comprises a comb, hair clips, hair clip plates, cord attachment points, and a frame. The comb was designed to optimize hair manageability prior to HAT application. The cord attachment point serves as an anchor, facilitating secure fixation of HADev to the scalp laceration site. Hair clips and plates collectively contributed to the secure fixation of hair strands before adhesive application (Figure 2). All components of HADev were constructed using standard‐grade polylactic acid (PLA) filament via 3D printing. Because this study was conducted as a simulation using a manikin model, the prototype device was used in a nonsterilized state.

### 2.2. Application Procedure of HADev

**FIGURE 3:**
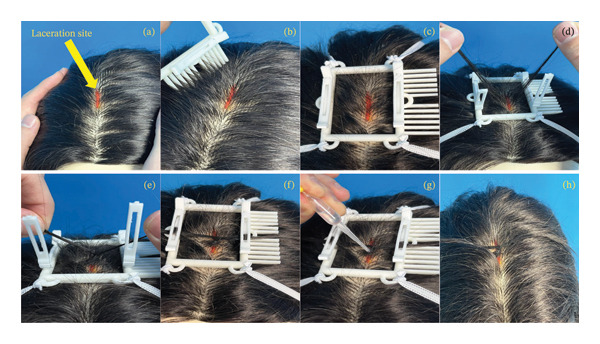
Steps involved in applying HADev.

Figure 3 illustrates the steps involved in applying HADev. Before initiating treatment, the scalp laceration site was confirmed (Figure 3(a)). Hair surrounding the scalp laceration site was trimmed using the integrated comb of the HADev (Figure 3(b)). The device was then secured to the scalp laceration site via the cord, which was connected to the designated cord attachment point. The cords were configured to encompass the posterior ear lobe, the base of the chin, the cheek, and the scalp. Within the rectangular framework of the HADev, each cord was anchored at a specific point and extended to the diametrically opposite attachment point (Figure 3(c)). To apply HAT, both sides of the hair clips were opened, and two hair strands were subsequently grasped to initiate the procedure (Figure 3(d)). A single twist was applied to approximate the wound edges (Figure 3(e)). Fixation of the twisted hair strands was achieved by closing both the hair clip and hair clip plate (Figure 3(f)). A single droplet of adhesive was applied to the twisted portion of the hair strands (Figure 3(g)). Following HADev removal, the twisted hair strands were secured, resulting in proper wound approximation (Figure 3(h)).

### 2.3. Study Design

This simulation study was conducted to evaluate the usability of HADev for manikins with long and short hair. This study was conducted at a tertiary teaching hospital in Gyeonggi Province, South Korea, between February and March 2022. The hospital serves as a regional level I emergency center and has an annual ED volume of approximately 45,000 patient visits.

### 2.4. Participants

A minimum anticipated sample size of 12 participants per group was calculated based on the findings of a previous study [12]. The final sample size was 20 participants, accounting for an anticipated dropout rate of 10%. Participants were recruited from the ED through an intrahospital announcement bulletin board, and all individuals provided written informed consent to participate in the study. The study participants included emergency physicians and emergency medicine residents with prior experience with HAT. Individuals were excluded from participation if they did not provide informed consent or if they presented with a musculoskeletal disease.

### 2.5. Study Protocol

A standardized operating procedure (SOP) was developed for the HADev application, including step‐by‐step instructions as illustrated in Figure 3. All participants followed the same protocol during the simulation. The SOP included the following: (1) wound site confirmation, (2) hair preparation using the integrated comb, (3) device fixation via cord attachment, (4) hair strand grasping and twisting, (5) clip closure, (6) adhesive application, and (7) device removal.

Participants were provided with a lecture on HADev by the researcher, lasting approximately 30 min, before commencing the simulation. A 30‐min practice period on a manikin was provided to each participant for familiarization with the HADev procedure. The participants were allowed two trials on the manikin for each hair length during the simulation. The manikin was fitted with wigs representing long (20‐cm) and short (8‐cm) hair, simulating typical male and female hair lengths, respectively.

The participants were randomly allocated to two groups: Group 1 (long hair–short hair sequence) and Group 2 (short hair–long hair sequence). Randomization was implemented using a lottery system, employing identical slips of paper for each group.

Following the group assignment, participants performed the HAT with HADev according to the allotted sequence. Procedure time was defined as the interval between the operator grasping the HADev and the subsequent removal of the HADev from the scalp of the manikin. Procedure time was recorded by a single, unblinded researcher (DKK) using a stopwatch. The participants were blinded to the time measurements. Success of the HADev procedure was defined as secure fixation of hair strands after HADev removal, whereas failure was defined as the inability to achieve secure fixation of hair strands or the inability to complete the procedure for any reason. A second attempt was permitted for participants who failed during the initial trial, with a 1‐min interval provided between the procedures.

All participants completed a brief questionnaire prior to the simulation to collect demographic data (age, sex, profession, and years of experience as an emergency medicine physician) and prior experience with HAT. Perceived difficulty and overall satisfaction were assessed using participants’ prior clinical experience with traditional HAT as a reference point. Specifically, participants were asked to retrospectively rate the difficulty and satisfaction of performing HAT in their previous clinical practice and then compare these ratings. Their recent simulation experience with HADev was compared against this reference. This comparison aimed to evaluate whether HADev offered improvements in ease of use and operator satisfaction relative to the conventional multioperator technique. Prior HAT experience was measured using a categorical variable with the following ranges: 0–10, 11–20, 21–30, 31–40, and > 40 times. Perceived difficulty and overall satisfaction with both the HAT procedure and the HADev device were evaluated using a 5‐point Likert scale, with difficulty rated from 1 (very easy) to 5 (very difficult) and satisfaction rated from 1 (excellent) to 5 (very poor).

### 2.6. Outcome Measures

The primary outcome was the success rate of HADev administration during the first attempt. Secondary outcomes included the procedure time for HADev when the procedure was successful and procedural failure, defined as the inability to complete the procedure due to any cause.

### 2.7. Statistical Analysis

Categorical variables are presented as counts and proportions, and comparisons were performed using the chi‐square test or Fisher’s exact test. Continuous variables are presented as either the median with interquartile range (IQR) or the mean with standard deviation, with comparisons performed using the *t*‐test or Mann–Whitney *U* test. Data analyses were performed using R software (Version 4.1.3; R Foundation for Statistical Computing). Statistical significance was set at a *p*‐value of < 0.05.

## 3. Results

**FIGURE 4:**
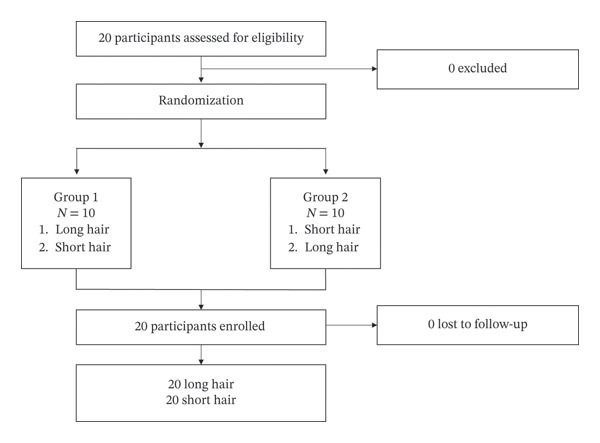
Flowchart of the simulation study.

Twenty participants were recruited for the study. The participants were randomly allocated to two groups of 10 participants each, and 40 HADev procedures were performed, with 20 for each hair length (Figure 4).

Among the participants, 13 (65.0%) were emergency medicine physicians and seven (35.0%) were residents. The median age was 34.5 years (IQR 30.5–42.5), and 3 participants (15%) were female. The median duration of work experience was 14.0 years (IQR 9.0–20.0) for physicians and 3.0 years (IQR 1.9–5.0) for residents. Regarding prior HAT experience, 45.0% of the HADev participants performed the procedure 0–10 times, followed by 25.0% who performed it 11–20 times. All residents (100%) had 0–10 years of experience, whereas physicians were distributed across all experience categories (Table 1).

**TABLE 1:** Characteristics of study participants.

Variables	All (*n* = 20)	Physician (*n* = 13)	Residents (*n* = 7)
Age (years), median (IQR)	34.5 (30.5–42.5)	37.0 (35.0–45.0)	30.0 (29.0–33.0)
Sex, *N* (%)			
Male	17 (85.0)	10 (76.9)	7 (100.0)
Female	3 (15.0)	3 (23.1)	0 (0.0)
Work experience (years), median (IQR)	9.0 (4.6–18)	14.0 (9–20)	3.0 (1.9–5)
HAT experience, *N* (%)			
0–10	9 (45.0)	2 (15.4)	7 (100.0)
11–20	5 (25.0)	5 (38.5)	0 (0.0)
21–30	2 (10.0)	2 (15.4)	0 (0.0)
31–40	2 (10.0)	2 (15.4)	0 (0.0)
> 40	2 (10.0)	2 (15.4)	0 (0.0)

Abbreviations: HAT, hair apposition technique; IQR, interquartile range.

For the short‐haired manikin, physicians and residents achieved success rates of 100% and 85.7%, respectively, with the HADev procedure. Median procedure times were 55.1 s (IQR 50.0–76.1) for physicians and 52.8 s (IQR 50.5–85.0) for residents. One procedural failure occurred in the resident group due to improper securing of hair to the HADev (Table 2).

**TABLE 2:** Outcomes of HADev procedure using short‐haired manikin.

Variables	All (*n* = 20)	Physician (*n* = 13)	Residents (*n* = 7)	*p*‐value
Success, *N* (%)	19 (95.0)	13 (100.0)	6 (85.7)	0.16
Time to success (seconds), median (IQR)	54.4 (50.3–77.8)	55.1 (50.0–76.1)	52.8 (50.5–85.0)	0.84
Cause of failure, *N* (%)				
Nonfixation	1 (5.0)	0 (0.0)	1 (14.3)	0.16

*Note:* HADev, hair apposition device.

Abbreviation: IQR, interquartile range.

For long‐haired manikins, physicians and residents achieved success rates of 92.3% and 100.0%, respectively, with the HADev procedure. Median procedure times were 63.0 s (IQR 57.7–68.1) for physicians and 62.1 s (IQR 55–78.8) for residents. One instance of procedural failure and device breakage occurred in the physician group (Table 3).

**TABLE 3:** Outcomes of HADev procedures using long‐haired manikin.

Variables	All (*n* = 20)	Physician (*n* = 13)	Residents (*n* = 7)	*p* value
Success, *N* (%)	19 (95.0)	12 (92.3)	7 (100.0)	0.45
Time to success (seconds), median (IQR)	62.5 (56.8–73.4)	63.0 (57.7–68.1)	62.1 (55.0–78.8)	0.97
Cause of failure, *N* (%)				
Breakage	1 (5.0)	1 (7.7)	0 (0.0)	0.45

*Note:* HADev, hair apposition device.

Abbreviation: IQR, interquartile range.

The median perceived difficulty of the HAT procedure was 2 (IQR 2–3) in both groups, whereas that of the HADev procedure was 1 (IQR 1–2). The median overall satisfaction with the HAT procedure was 2 (IQR 2–2) among physicians and 3 (IQR 2–3) among residents. The median satisfaction with HADev was 1 (IQR 1–2) in both groups (Table 4).

**TABLE 4:** Perceived difficulty and satisfaction scores for HAT and HADev procedures^∗^.

Variables	All	Physician	Residents	*p* value
HAT Difficulty^†^, median (IQR)	2 (2‐3)	2 (2‐3)	2 (2‐3)	0.82
HADev difficulty^†^, median (IQR)	1 (1‐2)	1 (1‐2)	1 (1‐2)	0.89
HAT satisfaction^‡^, median (IQR)	2 (2‐3)	2 (2‐2)	3 (2‐3)	0.35
HADev satisfaction^‡^, median (IQR)	1 (1‐2)	1 (1‐2)	1 (1‐2)	1.00

*Note:* HADev, hair apposition device.

Abbreviations: HAT, hair apposition technique; IQR, interquartile range.

^∗^HAT ratings reflect retrospective assessment of prior clinical experience, not concurrent comparison.

^†^Perceived difficulty of the HAT procedure (based on retrospective assessment of prior clinical experience) and HADev procedures (based on current simulation experience) was measured using a 5‐point Likert scale ranging from 1 (very easy) to 5 (very difficult).

^‡^Overall satisfaction with the HAT procedure (based on retrospective assessment of prior clinical experience) and HADev procedures (based on current simulation experience) was measured using a 5‐point Likert scale ranging from 1(excellent) to 5 (very poor).

### 3.1. User Error Analysis

During the 40 procedures, two types of procedural failures were observed: nonfixation (*n* = 1, 2.5%) and breakage (*n* = 1, 2.5%). Nonfixation occurred when the HADev frame became displaced, resulting in the wound not being centered within the frame. Breakage involved structural damage to the hair clip component during the opening process.

## 4. Discussion

In the current study, we assessed the applicability of the newly developed HAT prototype device, HADev. The device demonstrated a 95% success rate for both long‐ and short‐haired manikins. Median procedure times using the HADev were 62.5 s (IQR 56.8–73.4) for long hair and 54.4 s (IQR 50.3–77.8) for short hair.

HAT was developed in 2002 by Dr. Ong for the management of scalp lacerations and has been widely used owing to its simplicity, fewer complications, and cost‐effectiveness [9, 11]. However, the technique may require at least two medical personnel: one to maintain the twisted hair strands and the other to apply the adhesive. Recognizing the need for at least two medical personnel to perform HAT, we developed the HADev to enable single‐operator use. The device was designed to incorporate hair clips and hair clip plates, which securely hold the hair strands, thereby replacing the function of one medical staff member. Moreover, a comb was integrated to prepare the hair before initiating the HAT procedure, and cord attachment points were added to secure the HADev frame in the appropriate scalp position according to the laceration site. The operator retains the flexibility to use various types of cords to ensure secure fixation of the frame, contingent on the availability of resources within the ED. Thus, the HADev is anticipated to be useful in medical settings with limited resources and medical personnel for performing HAT.

The performance of HAT between physicians and nurses has previously been compared to evaluate the effectiveness, complications, and benefits of the procedure [13]. Ong et al. reported that trained nurses displayed safe HAT performance, achieving outcomes equivalent to those achieved by physicians. Similarly, we compared HADev performance between emergency medicine physicians and residents, assuming that they were specialists and novices in HAT, respectively. Despite the residents having less HAT experience, no statistically significant differences were observed between the two groups in perceived HAT difficulty, HADev difficulty, HAT satisfaction, and HADev satisfaction (Table 4). Moreover, the overall success rate was consistent across both long‐ and short‐hair conditions, with one procedure failure occurring in each hair type. No statistically significant difference in the procedure time was identified between the two groups in either scenario (Tables 2 and 3). These findings suggest that HADev can be readily used by healthcare workers with limited HAT experience following brief training. Future studies should evaluate HADev’s usability across a broader range of healthcare providers, such as nurses and emergency medical technicians, to ensure findings are generalizable across different user groups.

Based on the inherent characteristics of the HAT procedure, we hypothesized that the procedure time would be longer when performed on long hair. To the best of our knowledge, no previous studies have compared the procedure time for HAT under short‐ and long‐hair conditions. One possible explanation is that long hair can be trimmed to achieve a length conducive to optimal preparation for the HAT procedure. However, given the possibility of cosmetic dissatisfaction resulting from hair cutting, a comparative analysis of the procedure times for short and long hair was conducted. As anticipated, the median procedure time for long hair was approximately 10 s longer than that for short hair. During the simulation study, participants reported difficulty in extracting hair strands from the space between the HADev frame and the scalp, which may have contributed to the prolonged procedure time relative to the short‐hair condition. Structural modifications to HADev may be necessary to enhance its usability in future studies.

This study demonstrated median procedure times of 54.4 s (IQR 50.3–77.8) for short hair and 62.5 s (IQR 56.8–73.4) for long hair (Tables 2 and 3). In comparison, a prior study reported mean HAT procedure times of 9.0 min (standard deviation 5.6) for physicians and 12.8 min (standard deviation 7.5) for nurses [13]. Although a direct comparison of procedure times between the two studies was not feasible, the procedure time observed in the current study was substantially shorter than that reported previously. This discrepancy may be attributed to the use of a manikin instead of real patients in this study, which allowed operators to perform the procedure without the inherent pressure experienced in clinical settings. Furthermore, the manikin does not require any skin preparation procedures, such as disinfection or dressing. Considering the inherent differences in study design, future research should evaluate the application of HADev in real patients to evaluate its usability in clinical settings.

Two procedural failures were observed, including device breakage and nonfixation. Device breakage occurred during hair clip manipulation, specifically while opening the component. Nonfixation resulted from frame displacement leading to wound misalignment, as well as the failure of the hair clip and plate to interlock properly, leading to insufficient tension compared with manual hair traction during HAT. These failures may reflect both structural limitations of the prototype and operator inexperience. Future design improvements should focus on material durability, reinforcement of the hair clip hinge and locking mechanism, and enhanced frame stability to ensure precise alignment. Furthermore, incorporating human factors–oriented features, such as tactile or visual feedback, alongside increased operator proficiency in device handling, particularly in using the fingers to simultaneously twist hair and close the clip, will be essential to reduce procedural failures in clinical settings.

The prototype was fabricated using standard PLA via 3D printing to facilitate rapid design iteration and proof‐of‐concept testing [14]. While this approach was suitable for early development, it has inherent limitations in mechanical durability and sterilizability. In a clinical setting, the device must withstand mechanical stress during manipulation while maintaining consistent performance and sterility. To meet these requirements, a disposable single‐use system using certified medical‐grade materials would be required for clinical application. All components, including the comb, hair clips, hair clip plates, cord attachment points, and frame, would need to be fabricated from medical‐grade polycarbonate (PC) or medical‐grade PLA, with medical‐grade PC prioritized due to its superior mechanical strength, cost‐effectiveness, and suitability for mass production via injection molding. For clinical use, sterilization using ethylene oxide gas in accordance with ISO 11135 standards would be appropriate to ensure patient safety while preserving polymer integrity [15].

In a previous study, Ong et al. included patients with linear scalp wounds less than 10 cm in length and the presence of scalp hair at least 3 cm in length [9]. This inclusion criterion was established due to the inherent nature of the HAT technique, which requires a minimum hair length and a specific wound trajectory to maintain the physical tension created by the hair knots. This similar inherent nature also applies to our study. Because HADev was developed based on the HAT, its application is constrained in cases of nonlinear wounds. Regarding the minimum hair length requirement, the HADev frame has a width of approximately 6 cm; therefore, hair strands must be at least 4 cm long for proper application. This 4‐cm threshold is calculated based on half the device width plus a minimum of 1 cm required to secure the strand within the hair clip. Regarding wound length, we recommend a maximum length of 10 cm, consistent with the criteria used in the previous study. Although a wound may be linear, excessively long lacerations require greater hair knot tension. Because the HADev applies hair knots sequentially, wounds exceeding 10 cm may result in knot slippage or wound dehiscence. Further research is necessary to determine the optimal wound length for the secure application of the HADev.

## 5. Limitation

This study has several limitations. First, this manikin‐based simulation study represents a preliminary evaluation of human factors feasibility and does not provide evidence of clinical efficacy. Owing to the simulation‐based design, clinical outcomes could not be assessed. Therefore, further clinical studies are necessary before HADev can be implemented in real‐world clinical settings. Second, as HADev is a prototype device, further refinement is necessary to mitigate the risk of breakage or nonfixation. Third, procedure times were recorded by a single unblinded observer, which may have introduced observer bias. Given the exploratory, proof‐of‐concept nature of the study, the primary focus was on establishing basic device feasibility, and a comprehensive reliability assessment was beyond the scope of this study. Future research should incorporate enhanced measurement strategies, such as video‐based verification or multiple independent raters, to improve reliability. Fourth, this study was conducted at a single tertiary care center with a male‐predominant sample (85% male), limiting generalizability. The gender imbalance may not adequately represent the manual dexterity and ergonomic considerations of female healthcare providers. Participants consisted exclusively of physicians and residents, reflecting South Korean regulations that restrict wound closure to physicians. However, in many countries, other healthcare providers, such as nurses and emergency medical technicians, perform such procedures and would be primary HADev users. Our sample may not reflect the usability experience of these providers, who may differ in training and procedural approach. Future multicenter studies should include varied healthcare providers with balanced gender representation across different practice settings. Lastly, minor procedural steps varied slightly across participants, although an SOP was provided. Establishing uniform procedural implementation is essential to ensure the generalizability of HADev’s usability.

## 6. Conclusion

HADev, a prototype device developed for single‐operator HAT, was subjected to usability testing using both long‐ and short‐haired manikins. The device demonstrated feasibility for single‐operator use in a simulated environment, with a 95% success rate and procedure time of approximately 1 min for both hair conditions. Future research is necessary to enhance the usability of the device and evaluate its performance in clinical settings.

## Author Contributions

Dae Kon Kim participated in the conceptualization. Hyuksool Kwon performed the data curation. Hyun Soo Kim and Dae Kon Kim performed the formal analysis. Dongbum Suh was responsible for the funding acquisition. Dae Kon Kim conducted the investigation. Dae Kon Kim and Hyuksool Kwon performed the methodology. Dae Kon Kim and Dongbum Suh were responsible for the project administration. Jin Hee Lee was responsible for the resources. Jin Hee Lee and Hyoung Ju Lee performed the software. Dongbum Suh and Chulmin Ha were responsible for the supervision. Chulmin Ha and Hyoung Ju Lee performed the validation. Jin Hee Lee, Hyuksool Kwon, and Hyoung Ju Lee conducted the visualization. Hyun Soo Kim and Dae Kon Kim conducted the writing–original draft. Hyun Soo Kim, Dae Kon Kim, Dongbum Suh, and Chulmin Ha were responsible for the writing–review and editing.

## Funding

This study was supported by a research grant funded by the Seoul National University Bundang Hospital (No. 02‐2020‐0023).

## Disclosure

All authors contributed to editorial changes in the manuscript and read and approved the final manuscript.

## Ethics Statement

The Institutional Review Board of the Seoul National University Bundang Hospital approved this study with informed consent (No. B‐2108‐705‐301). The study participants provided written informed consent using the Seoul National University Bundang Hospital research consent form. All methods were carried out in accordance with relevant guidelines and regulations. This study has been performed in accordance with the Declaration of Helsinki.

## Conflicts of Interest

Dae Kon Kim and Dongbum Suh hold Korean Patent No. 10‐2639390 for HADev. Furthermore, a U.S. patent application for this invention is pending. Except for the two aforementioned authors, all other authors declare no conflicts of interest.

## Data Availability

The datasets used and/or analyzed during the current study are available from the corresponding author upon reasonable request.
